# Supplementary material for: Cardiovascular Risk across Glycemic Categories: Insights from a Nationwide Screening in Mongolia, 2022–2023
Source: J Clin Med. 2024 Oct 1;13(19):5866. doi: 10.3390/jcm13195866 (PMC11477117; doi:10.3390/jcm13195866)
Supplement: Supplementary file 1 [file jcm-13-05866-s001.zip › jcm-3201808-supplementary.pdf]

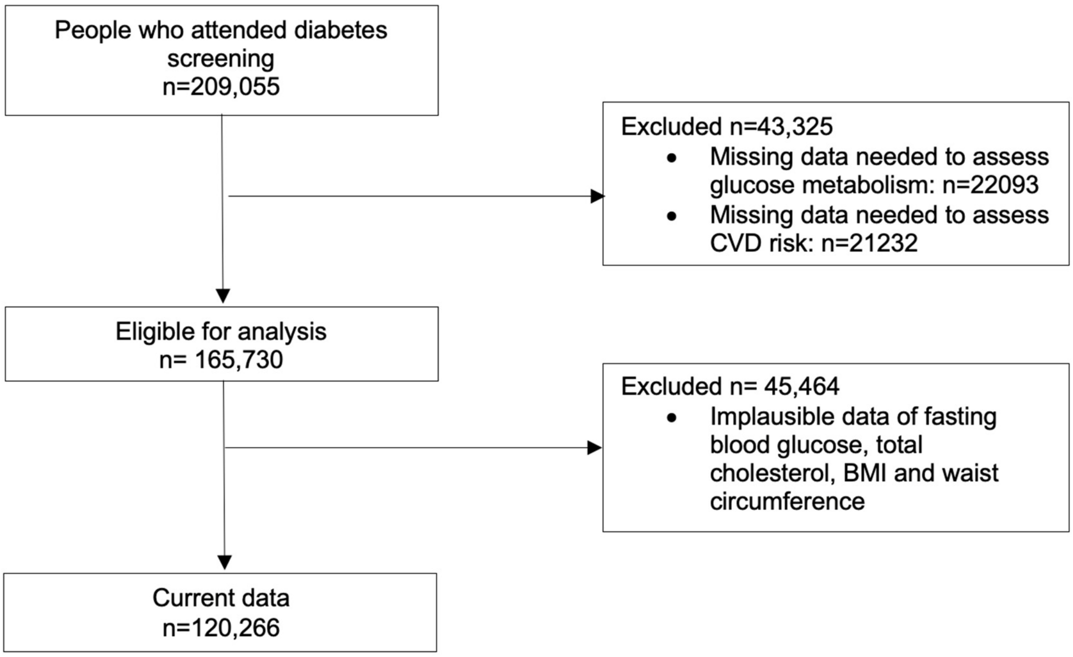

Supplementary Figure S1. Flowchart of the study population.

Supplementary Table S1. Prevalence of cardiovascular disease categories and ICD codes in the total population and in individuals with cardiovascular disease (CVD).

Category	ICD Codes	Number of cases	In total population (%)	In people with CVD (%)
Atherosclerosis	(ICD-10: I70)	54	0.04	0.12
Cardiac Arrhythmias	(ICD-10: I49)	1324	1.10	2.87
Conduction Disorders	(ICD-10: I44-I45)	553	0.46	1.20
Hypertension and Hypertensive Heart Disease	(ICD-10: I10-I15)	42354	35.22	91.86
Ischemic Heart Disease	(ICD-10: I20-I25)	2855	2.37	6.19
Other Cardiovascular Diseases	(ICD-10: I00-I99)	139	0.12	0.30
Pulmonary Heart Disease	(ICD-10: I26-I28)	64	0.05	0.14
Rheumatic Fever	(ICD-10: I00-I02)	364	0.30	0.79
